# Bluetongue Virus Surveillance in Yunnan, China, 2025: Isolation of Multiple Serotypes From *Culicoides* and Seasonal Seroepidemiology in Cattle

**DOI:** 10.1155/tbed/8145184

**Published:** 2026-07-15

**Authors:** Zhenxing Yang, Yuwen He, Yongkang Li, Teng He, Zhenxing Zhang, Jinxin Meng, Susheng Li, Nan Li, Haisheng Miao, Jianling Song

**Affiliations:** ^1^ Yunnan Tropical and Subtropical Animal Viral Disease Laboratory, Yunnan Animal Science and Veterinary Institute, Kunming, 650224, China; ^2^ College of Veterinary Medicine, Yunnan Agricultural University, Kunming, 650201, China, ynau.edu.cn; ^3^ Nujiang Animal Health Supervision Institute, Nujiang Lisu Autonomous Prefecture, Lushui, 533300, China

**Keywords:** bluetongue disease (BT), bluetongue virus (BTV), cattle, China, *Culicoides*, whole-genome sequences

## Abstract

Bluetongue (BT) virus (BTV) is an arthropod‐borne pathogen that causes substantial economic losses in ruminants globally. Yunnan Province, located in China’s tropical and subtropical border region, faces a high risk of BTV circulation. This study monitored BTV vectors, genetically characterized circulating BTV strains, and assessed seroprevalence to inform early warning efforts. In 2025, 46,000 *Culicoides* midges were collected from three counties, identified, and grouped into 460 pools. Additionally, 5934 cattle serum samples from 17 border counties were tested using C‐ELISA. Midge pools were screened by RT‐qPCR for BTV nucleic acids and then inoculated into C6/ 36 cells for virus isolation. Whole‐genome sequencing and phylogenetic analysis of VP2 and VP5 genes were performed on the isolates. *C. oxystoma* and *C. trithecoides* were the dominant midge species at all sites. RT‐qPCR identified 11 BTV‐positive midge pools, primarily involving *C. oxystoma*, *C. imicola*, *C. tainanus*, and *C. jacobsoni*. Four BTV isolates were identified as serotypes 1, 4, 5, and 16. Phylogenetic analysis of VP2 and VP5 showed all four strains likely belong to the Eastern topotype and are closely related to historical BTVs from China, Japan, India, and Australia. The overall BTV seroprevalence in cattle was 41.37% (95% confidence interval [CI], 40.12%−42.62%), with significantly higher rates in autumn (58.07%, 95% CI, 56.29%−59.85%) than in spring (24.87%, 95% CI, 23.32%−26.42%). Several BTV serotypes have been detected and isolated from diverse *Culicoides* in the border regions of Yunnan. Additionally, BTV seroprevalence has been observed in local cattle populations, indicating that this area may represent a significant BTV endemic hotspot. These findings underscore the need for ongoing vector surveillance and early warning systems to prevent BT outbreaks in susceptible livestock.

## 1. Introduction

Bluetongue (BT) virus (BTV) causes BT disease and is an arthropod‐borne virus classified within the genus *Orbivirus* of the family Reoviridae [1]. Certain *Culicoides* biting midges transmit it, infecting both domestic and wild ruminants, resulting in global agricultural losses estimated at up to US$ 3 billion [2]. Sheep are highly susceptible, and infection often results in severe symptoms or mortality [3]. In contrast, cattle and goats are typically asymptomatic or exhibit subclinical infections. However, cattle and goats infected with the Western topotype BTV‐8 strain in Europe show a higher incidence of clinical disease [4].

The BTV virion is composed of a triple‐layered capsid protein that encloses a genome of 10 double‐stranded RNA (dsRNA) segments that encode seven structural and four nonstructural proteins [5]. VP2 and VP5 are the capsid proteins of BTV and represent the most variable components, with their variability correlating with virus serotypes [6]. Analysis of VP2 gene sequences and results from virus tests have identified 36 distinct BTV serotypes isolated from livestock [7, 8]. Although structural proteins, except for VP2 and VP5, and nonstructural proteins are relatively conserved across BTV serotypes, their encoding sequences differ by geographic origin (topotype) of the isolates [9]. Most BTVs are classified as Eastern or Western topotypes and are further divided into geographic subgroups based on phylogenetic analysis of their nucleotide (nt) sequences [9].

Historically, BTV was confined to subtropical and tropical regions between latitudes 35°S and 50°N [10]. Its distribution has now expanded, with detections in Europe as far north as 60° latitude [11]. The most recent outbreak of BT, caused by BTV‐3, occurred in the Netherlands in 2023 and resulted in clinical signs and mortality in sheep and cattle [12]. Subsequently, BTV‐3 re‐emerged and spread rapidly across much of Europe, affecting more than 13 countries or regions. During this period, BTV‐12 was also identified for the first time in Europe [13]. The first reported BT outbreak in sheep in China occurred in Yunnan Province in 1979, with BTV‐1 (Y863) isolated from affected animals [14]. During the two national BTV surveillance programmers in China (1994–2000 and 2012–2018), 14 BTV serotypes (from BTV‐1 to 5, 7, 9, 12, 15, 16, 20, 21, 24, and 29) were isolated from sheep and cattle in southeastern and southwestern provinces, including Yunnan, Guangdong, Jiangsu, Hubei, and Guangxi provinces [9, 15, 16]. In the tropical regions of southwestern China, the BTV infection rate in cattle and goats ranges from 35% to 65% [17], indicating persistent transmission and the presence of efficient vectors.

Research on BTV vectors in China remains limited as most studies focus on detecting and isolating BTV from host animals. An early warning system is essential for detecting viral incursion and spread before clinical cases emerge. Vector monitoring is a key element in the rapid and accurate detection of arboviral activity. Yunnan is in southwest China, bordering Vietnam, Laos, and Myanmar. Much of the region lies within tropical and subtropical zones. A previous investigation conducted between 2012 and 2018 identified 13 BTV serotypes isolated from cattle and sheep in Yunnan [9, 15], indicating that this region is an ideal location for BTV monitoring. This study reports the detection of BTV in *Culicoides* specimens collected from three distinct regions along the Yunnan border. The whole‐genome of the virus was attempted to be obtained from *Culicoides* biting midges using virus isolation and next‐generation sequencing (NGS), and viral genomic characteristics and phylogenetic analysis were conducted. Furthermore, we examined the prevalence of BTV in cattle across the border regions of China, Laos, Myanmar, and Vietnam during both spring and autumn.

## 2. Materials and Methods

### 2.1. Collection and Sorting of *Culicoides* Specimens

**Figure 1:**
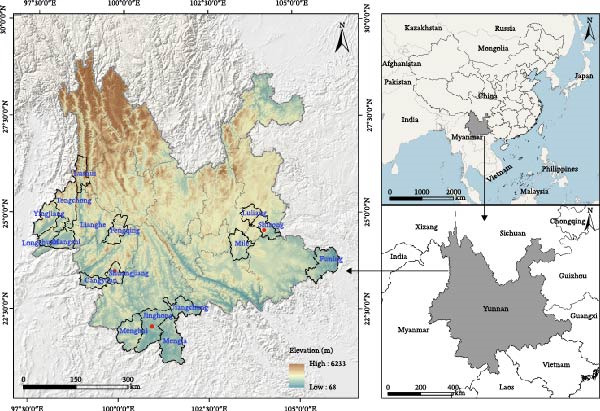
The geographical locations of *Culicoides* spp. and bovine serum sampling sites in Yunnan Province, China. The red dots 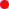 indicate *Culicoides* sampling sites, while those marked with black borders and blue place names are the 17 counties from which cattle serum was collected.

In 2025, *Culicoides* specimens were collected from cattle shelters in the suburbs of Shuangjiang, Jinghong, and Shizong Counties, Yunnan Province, using light traps (LTS‐M01, Wuhan Jixing Environmental Technology Co., Ltd., China) operated from 5 p.m. to 7 a.m. Two light traps were installed at separate cattle farms in each county and operated for one entire night to collect midges. These farm locations are situated near the borders with Myanmar, Laos, and Vietnam, respectively (Figure 1). We used the previously reported morphological identification atlas [18, 19] to identify *Culicoides* species preliminarily. Groups of 100 *Culicoides* specimens of the same species, collected from a single location, were pooled in a cryopreservation tube, stored in liquid nitrogen, and sent to the laboratory for subsequent analysis.

### 2.2. Serum Sample Collection

In spring and autumn of 2025, 5934 cattle serum samples were collected from 17 counties along the borders with Laos, Myanmar, and Vietnam. In Luliang and Lushui, cattle blood samples were collected in spring and autumn, respectively, from five randomly selected farms. In other locations, samples were collected in both seasons from 10 randomly selected farms. Each sample was collected from a different animal, aged 12–18 months. Blood was drawn from the jugular vein into tubes without an anticoagulant, and the tubes were allowed to clot undisturbed. Sample collection and serum separation were primarily conducted in collaboration with the local animal disease prevention and control center. Serum samples were collected in each county as follows: Cangyuan (*n* = 368), Fengqing (*n* = 368), Funing (*n* = 368), Jiangcheng (*n* = 300), Jinghong (*n* = 445), Lianghe (*n* = 368), Longchuan (*n* = 368), Luliang (*n* = 180), Lushui (*n* = 134), Mangshi (*n* = 368), Menghai (*n* = 368), Mengla (*n* = 368), Mile (*n* = 457), Shizong (*n* = 400), Shuangjiang (*n* = 320), Tencong (*n* = 368), and Yingjiang (*n* = 368) and sent to the laboratory under controlled temperature conditions. Currently, China has not administered any BTV vaccine, and cattle populations have not been immunized.

### 2.3. Nucleic Acid Extraction and BTV Detection

Each group of 100 *Culicoides* was homogenized in 1 mL of ice‐cold modified eagle medium (MEM) supplemented with 100 U/mL penicillin and 100 µg/mL streptomycin using a tissue grinder. A volume of 50 μL of the homogenized solution was used to extract viral nucleic acids using the MagMAX M‐96 Viral RNA Isolation kit and the MagMAX Express‐96 machine (Ambion, Thermo Fisher Scientific, USA). The extracted RNA was denatured at 95°C, quickly cooled on ice, and analyzed by RT‐qPCR with BTV‐specific primers [20] (forward: 5′—TGGAYAAAGCGATGTCAAA—3′, reverse: 5′—ACATCATCACGAAACGCTTC—3′) and probes (5′ FAM‐ARGCTGCATTCGCATCGTACGC‐BHQ1 3′). A 2 μL nucleic acid sample was mixed with 18 μL of the reaction solution prepared with the One Step PrimeScript RT‐qPCR Kit (Code Number RR064A, Takara Biotechnology Co., Ltd., Dalian) according to the manufacturer’s instructions. Positive and negative controls were included in each run. The positive control had a Ct value below 30, while the negative control showed no amplification. RT‐qPCR was performed on a Fast‐7500 Real‐Time PCR System (Applied Biosystems, Thermo Fisher Scientific, USA) with the following cycling conditions: 42°C for 5 min, 95°C for 10 s, followed by 40 cycles of 95°C for 10 s and 60°C for 34 s.

### 2.4. Cell Cultures and Viral Isolation

As previously described, *Aedes albopictus* (C6/36) cells were utilized for viral isolation [21]. C6/ 36 cells were maintained at 28°C with 5% CO_2_ in a medium composed of equal parts Dulbecco’s MEM (DMEM) and Roswell Park Memorial Institute 1640 (RPMI) medium, supplemented with 10% fetal bovine serum (FBS). Homogenized *Culicoides* samples were centrifuged at 10,000 × *g* for 2 min at 4°C and then filtered through a 0.22 µm filter unit (Merck Millipore Ltd., Cork, Ireland). A volume of 100 µL of the resulting filtrate was then inoculated onto C6/ 36 cell monolayers in 6‐well plates. The cells were observed daily for cytopathic effects (CPEs) across three passages. After observing CPEs in the cultures, we collected the supernatants and stored them at −80°C for subsequent analysis.

### 2.5. Amplification and Sequencing of Full‐Length Complementary DNA (cDNA)

Total RNA was extracted from virus‐infected cells using a RNAiso Plus (Takara Biotechnology Co., Ltd., Dalian). Viral RNA was extracted from total RNA utilizing the 2 M LiCl protocol, as outlined by Attoui et al. [22]. The dsRNA was subsequently reverse transcribed into cDNA using the full‐length amplification of cDNA (FLAC) technique, as previously described [23, 24]. Viral cDNA samples were sent to the company (Magigene Biotechnology Co., Ltd., Guangdong) for NGS. Following read preparation, sequence verification, and data filtering, we performed de novo assembly with Abyss (v2.0.2) [25] and SOAPdenovo (v2.04) [26] to construct genomic segment sequences for each isolate.

### 2.6. Molecular Phylogenetic Analysis

Reference sequences for BTVs were retrieved from GenBank on February 1, 2026 and are presented in Supporting Information 1: Table S1. We identified open reading frames (ORFs) in viral genomic segments and translated them into amino acid (aa) sequences using the ORFfinder (https://www.ncbi.nlm.nih.gov/orffinder/). Consensus sequence alignments were performed using MAFFT (v7.520) [27], and nt and aa identities were determined with BioAider (v1.627). The best‐fit models for maximum likelihood (ML) trees were identified using ProtTest 3.4.2 and MEGA‐11. The GTR + G + I model was applied to VP2 and the TN93 + G + I model to VP5. Phylogenetic trees and pairwise distances were performed in MEGA‐11 using the *p*‐ distance parameter with 1000 bootstrap replicates.

### 2.7. Serological and Statistical Analyses

We employed the ID Screen Bluetongue Competition ELISA kit (IDVet, Grabels, France) to detect anti‐BTV antibodies in cattle serum samples. Tests were performed according to the manufacturer’s instructions using the recommended cut‐off values. The 95% confidence interval (CI) were estimated using the Agresti‐Coull method. Epidemiologic data from different seasons served as independent variables, while BTV serological test results were the dependent variables. All independent variables were analyzed through cross‐tabulations and descriptive statistics, including frequencies and percentages. These variables were screened against the response variable ( ELISA ) using a chi‐square test (*χ* ^2^). Epidemiologic data were employed to develop binary logistic models to estimate the odds ratio (OR) for BTV seropositivity. Differences with *p*‐ values below 0.05 were considered statistically significant, while those below 0.01 were considered highly significant. Analyses were performed using SPSS Version 22.0.

## 3. Results

### 3.1. *Culicoides* Collection

We collected 46,000 *Culicoides* samples from three sites, representing nine species and the subgenus *C. trithecoides* (Table 1). The composition of the dominant species varied by location. In Jinghong, *C. oxystoma* (51.5%), *C. trithecoides* (18.9%), and *C. jacobsoni* (14.4%) were the most abundant among the collected *Culicoides*. In Shizong, *C. trithecoides* (26.5%), *C. tainanus* (25.4%), and *C. oxystoma* (17.5%) were the dominant species. In Shuangjiang, *C. oxystoma* (30.6%), *C. trithecoides* (29.4%), and *C. arakawae* (15.9%) were the primary identified species. *C. oxystoma* (35.36%) and *C. trithecoides* (24.52%) were the most common species across all three sampling sites. Conversely, *C. orientalis* (1.98%), *C. sumatrae* (2.68%), *C. actoni* (0.13%), and *C. asiana* (0.08%) had the lowest proportions. To support our research, we divided the 46,000 *Culicoides* samples into 460 pools, classified by nine morphologically identified species and mixed species from the subgenus *C. trithecoides* without distinguishing between blood‐fed and unfed individuals. We extracted viral nucleic acids from homogenized pool liquids and inoculated them onto C6/ 36 cells to isolate BTV.

**Table 1:** *Culicoides* biting midges collected from the suburbs of Jinghong, Shizong, and Shuangjiang counties in Yunnan Province, China.

Geographic origin (county)	Longitude and latitude	Altitude (m)	Collection date	Number (proportion, %) of different species										Total
				*C. arakawae*	*C. oxystoma*	*C. imicola*	*C. tainanus*	*C. jacobsoni*	*C. orientalis*	*C. sumatrae*	*C. actoni*	*C. asiana*	^a^ *C. trithecoides*	
Jinghong	100°51′45″ E, 22°5′20″ N	740	June 12, 2025	900 (5%)	9270 (51.5%)	1134 (6.3%)	486 (2.7%)	2583 (14.4%)	234 (1.3%)	0	0	0	3393 (18.9%)	18,000
Shizong	104°17’28″ E, 24°36’33″ N	937	June 27, 2025	540 (4.5%)	2100 (17.5%)	432 (3.6%)	3048 (25.4%)	1272 (10.6%)	612 (5.1%)	720 (6.0%)	60 (0.5%)	36 (0.3%)	3180 (26.5%)	12,000
Shuangjiang	99°48’28″ E, 23°30’14″ N	995	July 16, 2025	2544 (15.9%)	4896 (30.6%)	1520 (9.5%)	1120 (7.0%)	640 (4.0%)	64 (0.4%)	512 (3.2%)	0	0	4704 (29.4%)	16,000
Total				3984 (8.66%)	16,266 (35.36%)	3086 (6.71%)	4654 (10.12%)	4495 (9.77%)	910 (1.98%)	1232 (2.68%)	60 (0.13%)	36 (0.08%)	11,277 (24.52%)	46,000

^a^ *C. trithecoides* refers to specimens in this subgenus with a yellow scutum, such as *C. flavescens*, *C. fordae*, and *C. palpifer*.

### 3.2. RT‐qPCR Detection of BTV in *Culicoides*

A total of 460 pools of conspecific *Culicoides*, representing nine species and one subgenus, were tested using RT‐qPCR for BTV. Of the sample pools, 417 showed no reaction. Forty‐three pools had Ct values below 35; of these, 32 had values between 30 and 35 and were classified as weakly positive. Only 11 pools (2.39%) had Ct values under 30, considered strongly positive, suggesting a possible BTV infection (Table 2). Morphologically, these 11 positive samples were identified as *C. oxystoma* (4 pools), *C. imicola* (1 pool), *C. tainanus* (4 pools), and *C. jacobsoni* (2 pools). *C. oxystoma* was collected from Jinghong and Shuangjiang; *C. jacobsoni* from Jinghong; *C. tainanus* from Shizong and Shuangjiang; and *C. imicola* from Shuangjiang. In Jinghong, *C. oxystoma* and *C. jacobsoni* accounted for ~51.5% and 14.4% of the total catch, respectively. In Shizong, *C. tainanus* made up around 25.4%. In Shuangjiang, catches included about 30.6% *C. oxystoma*, 9.5% *C. imicola*, and 7.0% *C. tainanus* (Table 1).

**Table 2:** Detection of BTV nucleic acid by RT‐qPCR in individual specimens of *Culicoides* species collected from Yunnan Province, China.

Species	Source^a^	Number of pools tested	RT‐qPCR test results^b^ (values are the number of pools)				Percentage of strongly positive pools (Ct≤30)
			Ct≤25	25＜Ct≤30	30＜Ct≤35	Undetected	
*C. arakawae*	JH, SZ, SJ	40	0	0	3	37	0
*C. oxystoma*	JH, SZ, SJ	163	1	3	7	152	2.45%
*C. imicola*	JH, SZ, SJ	30	0	1	4	25	3.33%
*C. tainanus*	JH, SZ, SJ	46	1	3	4	38	8.70%
*C. jacobsoni*	JH, SZ, SJ	45	1	1	5	38	4.44%
*C. orientalis*	JH, SZ, SJ	9	0	0	2	7	0
*C. sumatrae*	SZ, SJ	12	0	0	0	12	0
*C. actoni*	SZ	1	0	0	0	1	0
*C. asiana*	SZ	1	0	0	0	1	0
*C. trithecoides*	JH, SZ, SJ	113	0	0	7	106	0
Total		460	3	8	32	409	2.39%

^a^JH, Jinghong; SZ, Shizong; SJ, Shuangjiang.

^b^A Ct value between 35 and 30 indicates possible positivity, classified as weakly positive. A Ct value below 30 is considered indicative of infection positivity and is classified as strongly positive.

### 3.3. Virus Isolation and Identification

The 11 pools of BTV‐positive samples were filtered and inoculated into C6/ 36 cells. Four isolates caused strong CPEs in C6/ 36 cells after 72 h during the third blind passage. These four isolates were confirmed as BTV by RT‐qPCR with Ct values below 20. Next, BTV serotypes were determined using serotype‐specific RT‐qPCR with 12 pairs of primers and probes (Supporting Information 2: Table S2) [28]. The four BTVs were identified as serotypes 1, 4, 5, and 16. The genomes of these isolates were amplified using the FLAC technique and sequenced by NGS, and the associated GenBank accession numbers are listed in Table 3. Subsequently, serotype‐specific RT‐qPCR for BTV‐1, ‐4, ‐5, and ‐16 was used to test the 11 BTV‐positive midge pools. In Jinghong, all *C. oxystoma* and *C. jacobsoni* samples tested positive for BTV‐1 except for one *C. jacobsoni* sample that tested positive for BTV‐5. In Shizong, all *C. tainanus* samples tested positive for BTV‐4. In Shuangjiang, all three *Culicoides* species, *C. oxystoma*, *C. imicola*, and *C. tainanus*, were positive for BTV‐16.

**Table 3:** Details of four viruses isolated from *Culicoides* collected in Jinghong, Shizong, and Shuangjiang counties, Yunnan Province, China.

Isolates^a^	Identification	Location	Hosts	GenBank accession number
JH25C064	BTV‐1	Jinghong	*C. oxystoma*	PZ244762–PZ244771
JH25C132	BTV‐5	Jinghong	*C. jacobsoni*	PZ244772–PZ244781
SZ25C057	BTV‐4	Shizong	*C. tainanus*	PZ244792–PZ244801
SJ25C081	BTV‐16	Shuangjiang	*C. imicola*	PZ244782–PZ244791

^a^JH, SZ, and SJ denote the midge collection sites of Jiangcheng, Shizong, and Shuangjiang, respectively; the number 25 refers to 2025, the year samples were collected; C stands for *Culicoides*, and the following number indicates the serial number of the midge sample pool.

### 3.4. Genome Characteristics of the Four Strains

Details of each genomic segment for the four isolates (including the encoded protein length) are shown in Supporting Information 3: Table S3. The genome lengths of JH25C064, JH25C132, SZ25C057, and SJ25C081 are 19,188, 19,173, 19,177, and 19,187 bp, with G+ C contents of 43.37%, 43.74%, 43.69%, and 43.37%, respectively. The segment lengths among the four isolates are highly conserved, except for Seg‐2, which are 2940, 2921, 2926, and 2935 bp and encode 961, 955, 956, and 959 aas, respectively. Analysis of the 5′ and 3′ noncoding regions (NCRs) showed that all segments of the four isolates possess highly conserved terminal nt sequences at the 5′ and 3′ NCRs (5′—GUUAAAA and ACUUAC—3′), except Seg‐3, which contains GUUAAAT at the 5′ NCR. The 5′ and 3′ NCRs of the four isolates accounted for 3.72%−3.75% of their total genome sizes.

### 3.5. Sequence and Phylogenetic Analysis of Seg‐2/VP2

**Figure 2:**
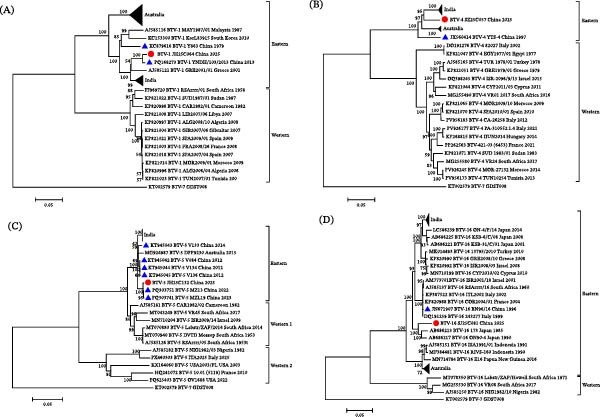
Phylogenetic analysis of VP2 genome coding sequences from four isolates and their homologous BTV serotypes: BTV‐1 (A), BTV‐4 (B), BTV‐5 (C), and BTV‐16 (D). The ML tree was constructed in MEGA 11 using the GTR + G + I model under the Bayesian information criterion (BIC), with 1000 bootstrap replicates. Each reference BTV strain is labeled as “GenBank accession number_ Serotype_ Strain number_ Country_ year of isolation.” Outgroup viruses are labeled as “GenBank accession number_ Serotype_ Strain number.” Red dots 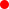 indicate isolates from this study, and blue triangles 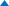 indicate other BTV isolates from China.

To confirm the serotypes of the four isolates, we conducted sequence and phylogenetic analyses of their Seg‐2, which identified them as serotypes 1, 4, 5, and 16, respectively. The phylogenetic analysis indicates that the four BTVs cluster with their respective homologous serotypes, while strains from China, India, Australia, and Japan are grouped within the Eastern topotypes (Figure 2). In the phylogenetic trees, BTVs are divided into two major topotypes, Western and Eastern, based on geographic origin. However, in the phylogenetic tree of BTV‐5, strains from Cameroon, South Africa, and Israel form one branch (Western 1). In contrast, strains from Nigeria, Italy, France, and the USA form a separate branch (Western 2).

The VP2 of JH25C064 is most closely related to BTV‐1 (YNDH/103/2013), isolated in China in 2013, with nt and aa sequence identities of 98.82% and 99.06%. In comparison, its identities with another Chinese BTV‐1 strain (Y863) are 90.78% (nt) and 96.26% (aa). The nt and aa identities between JH25C064 and Eastern topotype strains ranged from 86.66% to 98.82% (*μ* = 89.98%) and 93.45% to 99.06% (*μ* = 95.15%), respectively. For Western topotype strains, nt and aa identities ranged from 73.94% to 74.32% (*μ* = 74.13%) and 81.81% to 82.33% (*μ* = 82.06%), respectively.

The VP2 nt and aa identities between SZ25C057 and the BTV‐4 strain (YTS‐2) from China (1997) were 91.85% and 97.18%, respectively. The identities with Eastern topotype strains ranged from 91.85% to 93.56% for nt (*μ* = 92.70%) and from 95.92% to 97.18% for aa (*μ* = 96.55%). In contrast, the identities with Western topotype strains ranged from 73.01% to 74.45% for nt (*μ* = 73.75%) and from 80.83% to 81.19% for aa (*μ* = 80.83%).

The VP2 nt and aa between JH25C132 and Chinese BTV‐5 strains ranged from 98.15% to 99.72% (*μ* = 98.87%) and 98.54%–99.58% (*μ* = 99.01%), respectively. Compared to the Eastern topotype strains, identities ranged from 97.94% to 99.72% (*μ* = 98.28%) for nt and from 98.54% to 99.58% (*μ* = 98.8%) for aa. In contrast, identities with Western‐1 and Western‐2 strains were lower: 93.88%–95.47% (*μ* = 94.42%) and 76.85%–77.82% (*μ* = 77.44%) for nt and 95.71%–97.38% (*μ* = 96.84%) and 85.88%–87.66% (*μ* = 86.84%) for aa, respectively.

The VP2 of SJ25C081 shows the highest similarity to the Japanese BTV‐16 strain (ON90‐4), with nt and aa identities of 95.83% and 98.23%, respectively. Its identity to the Chinese strain (BN96/16) is slightly lower at 94.51% (nt) and 97.71% (aa). Identities with Eastern topotype strains range from 88.20% to 95.83% for nt (*μ* = 92.24%) and 91.98% to 98.23% for aa (*μ* = 96.45%). In contrast, identities with Western topotype strains are lower, ranging from 73.78% to 74.31% for nt (*μ* = 74.12%) and 81.67% to 83.23% for aa (*μ* = 82.5%).

### 3.6. Sequence and Phylogenetic Analysis of Seg‐6/VP5

**Figure 3:**
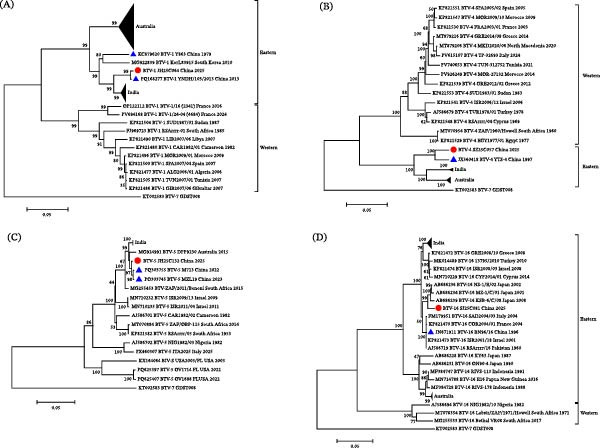
Phylogenetic analysis of VP5 genome coding sequences from four isolates and their homologous BTV serotypes: BTV‐1 (A), BTV‐4 (B), BTV‐5 (C), and BTV‐16 (D). The ML tree was constructed in MEGA 11 using the TN93 + G + I model under the BIC, with 1000 bootstrap replicates. Each reference BTV strain is labeled as “GenBank accession number_ Serotype_ Strain number_ Country_ year of isolation.” Outgroup viruses are labeled as “GenBank accession number_ Serotype_ Strain number.” Red dots 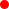 indicate isolates from this study, and blue triangles 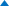 indicate other BTV isolates from China.

The VP5 phylogenetic tree exhibits a pattern like that observed in VP2. In all phylogenetic trees, the four isolates analyzed in this study cluster with isolates from China, Japan, India, and Australia (Figure 3). Subtle differences were also observed. The Western and Eastern topotypes are clearly distinguished in the phylogenetic trees of BTV‐1, BTV‐4, and BTV‐16 but not in those of BTV‐5.

VP5 of JH25C064 has the highest sequence identity with the BTV‐1 strain (YNDH/103/2013), isolated in China in 2013, with nt and aa identities of 99.56% and 99.81%, respectively. Comparisons between JH25C064 and Eastern topotype strains show nt identities ranging from 88.74% to 99.56% (*μ* = 91.34%) and aa identities from 97.53% to 100% (*μ* = 98.42%). In contrast, identities with Western topotype strains range from 77.42% to 79.32% (*μ* = 78.25%) for nt and from 94.69% to 95.45% (*μ* = 95.14%) for aa.

The VP5 of SZ25C057 still exhibits the highest similarity to the BTV‐4 strain (TYS‐4), with nt and aa identities of 95.58% and 99.81%, respectively. The identities between SZ25C057 and the Eastern topotype strains range from 90.45% to 96.58% (*μ* = 91.37%) for nt and from 98.67% to 99.81% (*μ* = 98.92%) for aa. These values are significantly higher than those observed with the Western topotype, which range from 78.87% to 79.57% (*μ* = 79.31%) for nt and from 92.98% to 93.74% (*μ* = 93.42%) for aa.

The VP5 of JH25C132 shows the highest sequence identity to strains from China, India, and Australia, with nt identities of 99.56%, 98.88%, and 98.57% and aa identities of 100%, 99.81%, and 99.62%, respectively. It is also closely related to the South African strain, with nt identity ranging from 95.64% to 98.55% (*μ* = 96.55%) and an aa identity of 99.43%. The most distant strains are from Nigeria, Italy, and the USA, with nt identities ranging from 86.59% to 89.56% (*μ* = 87.8%) and aa identities from 97.34% to 99.24% (*μ* = 98.33%).

The VP5 of SJ25C081 exhibits the highest genetic similarity to the Japanese strains NS‐1/E/02, MZ‐1/C/01, and KSB‐6/C/08, with nt identities ranging from 98.17% to 98.48% (*μ* = 98.29%) and aa identities from 99.05% to 100% (*μ* = 99.62%). In contrast, the identities to the Chinese strain BN96/16 are slightly lower at 97.72% (nt) and 99.62% (aa). Sequence identities with Eastern topotype strains range from 91.52% to 98.48% at the nt level (*μ* = 95.16%) and from 98.67% to 100% at the aa level (*μ* = 99.34%). In comparison, sequence identities with Western topotype strains are lower, ranging from 79% to 97.57% (*μ* = 79.21%) for nt and from 95.07% to 95.45% (*μ* = 95.26%) for aa.

### 3.7. Epidemiological Investigation of BTV in Cattle in Yunnan

**Figure 4:**
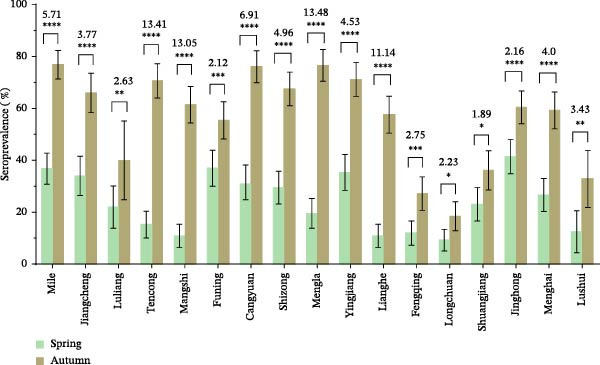
Seroprevalence of BTV antibodies in cattle serum detected by C‐ELISA across 17 counties in Yunnan Province, China. Error bars represent the 95% confidence interval (CI) for seroprevalence.  ^∗^, ^∗∗^,  ^∗∗∗^, and  ^∗∗∗∗^ indicate *p*‐ values less than 0.05, 0.01, 0.001, and 0.0001, respectively. The numbers above the asterisks ( ^∗^) indicate the odds ratio (OR) derived from the chi‐square test comparing spring and autumn.

To further evaluate BTV dispersal, 5934 cattle serum samples were collected from 17 counties in Yunnan Province, which borders Vietnam, Laos, and Myanmar (Figure 1), and analyzed for BTV antibodies using C‐ELISA. Among all samples tested, 2455 (41.37%, 95% CI 40.12%–42.62%) were seropositive for BTV antibodies. Significant differences were observed in serum test results between spring and autumn. The annual seroprevalence of BTV in cattle increased progressively from spring to autumn. The highest prevalence of BTV antibodies was observed in autumn (58.07%, 95% CI 56.29%−59.85%), whereas the lowest was observed in spring (24.87%, 95% CI 23.32%−26.42%). An analysis of test results from various regions demonstrates that, except for spring and autumn samples from Shuangjiang and Longchuan, detection results in all other regions between the two seasons are highly statistically significant (Figure 4). In particular, ORs comparing spring and autumn test results exceeded 10 in Tengchong, Mangshi, Mengla, and Lianghe and exceeded 5 in Mile and Cangyuan. BTV seroprevalence rates vary by region. In spring, cattle serum samples tested positive at rates below 40% in all regions except Jinghong. In particular, the seroprevalence rates in Longchuan, Fengqing, Lushui, Lianghe, and Mangshi were all below 15%. In autumn, the BTV seroprevalence in all regions except Fengqing, Longchuan, Shuangjiang, and Lushui exceeded 40%, with rates exceeding 70% in Mile, Tengchong, Cangyuan, Mengla, and Yingjiang.

## 4. Discussion

Between 1994 and 2018, two national surveillance projects identified 13 BTV serotypes (1–5, 9, 11, 12, 15, 16, 21, 23, and 24) in Yunnan Province [16, 17]. After this period, systematic monitoring of BT in Yunnan was largely discontinued. In 2023, BTV‐3 was first detected in the Netherlands and subsequently spread rapidly across several European countries, significantly affecting local livestock industries [12, 13]. To assess the current prevalence of BTV in Yunnan Province, particularly in border areas, this study systematically identified *Culicoides* species and tested samples from Shuangjiang, Jinghong, and Shizong counties for BTV. A seroepidemiological investigation was also conducted among cattle herds along the borders with Myanmar, Laos, and Vietnam. The findings confirmed the diversity of *Culicoides* populations in Yunnan and resulted in the successful isolation of four BTV serotypes (1, 4, 5, and 16) from these *Culicoides*. Serological testing of 5934 cattle serum samples from 17 counties indicated an overall BTV seroprevalence of 41.37% (95% CI: 40.12%−42.62%), with a significantly higher rate observed in autumn compared to spring. These results provide new insights into the current prevalence, viral diversity, and transmission risks of BTV in the region.

*Culicoides* are primary vectors for BTV, and the spread of BT is closely tied to their species composition and distribution. While there are 1,400 *Culicoides* species globally, only a small subset transmits BTV [29]. To date, 168 species of *Culicoides* have been identified in Southeast Asia, with more than 70 species recorded in Yunnan Province, China [30]. In this study, we collected nine species and one subspecies (*C. trithecoides*). *C. oxystoma* and *C. trithecoides* were the dominant species, followed by *C. tainanus*, *C. jacobsoni*, *C. arakawae*, and *C. imicola*. These species are prevalent and widely distributed on livestock farms in southern Yunnan and throughout southern Asia [31]. BTV was detected and isolated from four species: *C. oxystoma*, *C. imicola*, *C. tainanus*, and *C. jacobsoni*, indicating that multiple local *Culicoides* species may be involved in BTV maintenance and transmission. Among these, *C. oxystoma* was the dominant species across all three sampling sites and was detected in positive pools in Jinghong and Shuangjiang, suggesting that it may be a potential vector species for BTV transmission in this region. *C. oxystoma* is recognized as a potential vector for BTV [29, 31], and this study offers additional virological evidence supporting its vector competence. Additionally, *C. imicola* is the primary vector for BTV in Europe and Africa [29]. This species was found at all three sampling sites in this study, and BTV was detected from specimens collected in Shuangjiang. Although detection rates (3.33%) were low, ongoing monitoring and further research into the population dynamics and transmission efficiency of this species are needed.

Distinct serotype distribution patterns were observed across regions: BTV‐1 and BTV‐5 in Jinghong, BTV‐4 in Shizong, and BTV‐16 in Shuangjiang. This aligns with previously monitored BTV serotypes in China, especially in Yunnan and Guangdong [15–17]. Notably, two BTV serotypes were isolated in Jinghong, while Jinghong and Shuangjiang, though only 200 km apart, have different prevalent serotypes. This confirms the diversity of BTV serotypes in the region and suggests relatively independent transmission cycles in different border areas, likely influenced by vector distribution, livestock movement, and cross‐border connectivity. These findings enhance our understanding of the BTV vector spectrum in Yunnan and support the hypothesis that Culicoides is a key transmission link in the local epidemic cycle.

Phylogenetic analysis of Seg‐2/VP2 and Seg‐6/VP5 showed that all four isolates clustered within the Eastern topotype and were more closely related to strains previously reported from China, Japan, India, and Australia. The nt and aa identities for Seg‐2/VP2 exceeded 86.66% and 91.98%, respectively, while those for Seg‐6/VP5 exceeded 88.74% and 97.53%, respectively. However, these four strains differ significantly from the Western topotype of European and African strains in Seg‐2/VP2 and Seg‐6/VP5. Seg‐2/VP2 identities are below 77.82% and 87.66%, while Seg‐6/VP5 identities are below 89.56% and 99.24%, respectively. The results demonstrate that the Seg‐2/VP2 and Seg‐6/VP5 gene segments of these strains are more closely related to those of Asian strains than to those of Western topotype strains. These strains likely belong to the Eastern topotype, but further analysis of additional sequence fragments is needed for confirmation. This observation also indicates the cocirculation of multiple BTV serotypes in the border regions of Yunnan. However, the presence of multiple serotypes in the same geographic area enables genomic segment exchange, potentially resulting in new, more virulent reassortants. Continued monitoring is essential.

Serological surveillance of 5934 cattle serum samples from 17 counties in Yunnan Province showed an overall BTV seropositivity rate of 41.37% (95% CI 40.12%−42.62%), which falls within the 35%–65% range previously reported in southern China [17, 32], indicating that BTV infection is very common among local cattle herds. Since the BTV vaccine has not been used in China, the seroprevalence rate reflects exposure to natural infection. This confirms that cattle may be the primary animal hosts and that subclinical infection is ongoing. Moreover, BTV seroprevalence reached its highest levels in autumn and was lowest in spring. The differences between these two seasons were highly statistically significant in most regions. In Tengchong, Mangshi, Mengla, and Lianghe, the OR increased by more than tenfold from spring to autumn. This pattern corresponds to the seasonal dynamics of *Culicoides* activity. Density remains low during the cooler, drier winter and spring but peaks in the warmer, more humid summer and autumn [33, 34], thereby significantly increasing the risk of virus transmission between hosts and vectors. Additionally, seroconversion in animals is characterized by a lag period. Consequently, animals infected with the virus during periods of high vector density in summer may test seropositive in autumn, contributing to increased positivity rates observed in autumn. These findings align with previous serological investigations of Tibet *orbivirus* (TIBOV), an orbivirus also transmitted by *Culicoides*. Before July, all 13 sentinel cattle in Yunnan tested negative for TIBOV ; by September, all animals tested positive [35]. This discovery underscores the need to enhance monitoring of the *Culicoides* population and BTV transmission dynamics in summer and autumn. Ongoing cross‐border collaboration in monitoring vectors and hosts is especially important to prevent the nationwide spread through Yunnan.

This study has several limitations. First, the limited number of sampling points and midges may not fully represent all potential BTV vector species or their infection status across the Yunnan border area. Second, while the virus was monitored and isolated from *Culicoides*, experimental infection studies are still needed to confirm the vector efficiency of these species. Third, frequent reassortment means that the evolutionary history of complete BTV genomes cannot be represented by a single phylogenetic tree [36, 37], and this study did not perform systematic phylogenetic or recombination analyses of other gene segments (except Seg‐2 and Seg‐6) across these four strains. Finally, the serological survey was limited to indicating past infection and was unable to identify specific infection serotypes or determine the timing of infection. Furthermore, the relatively small number of farms and serum samples collected may have led to clustering within farms and regions, potentially overestimating the statistical confidence.

## 5. Conclusions

In 2025, four BTV serotypes (1, 4, 5, and 16) were isolated from *Culicoides* in the border areas of Yunnan (Jinghong, Shizong, and Shuangjiang), confirming a complex transmission cycle involving multiple serotypes. Sequence and phylogenetic analyses of Seg‐2/VP2 and Seg‐6/VP5 indicate that these four isolates may belong to the Eastern topotype and are closely related to Asian strains. *C. oxystoma* was the dominant species, and BTV was detected in several locations, suggesting that it may be a potential vector species in this region. BTV was also detected in *C. imicola*, *C. tainanus*, and *C. jacobsoni*, indicating that several *Culicoides* species may be involved in virus transmission. The BTV positivity rate in cattle serum during autumn is significantly higher than in spring, with the OR exceeding 4 compared with spring and surpassing 10 in certain regions. This trend is likely related to peak *Culicoides* activity in summer and autumn as increased *Culicoides* density leads to higher infection rates among cattle. These findings provide essential data for the early warning of BT in southwestern China.

## Author Contributions

Jianling Song and Zhenxing Yang conceived and designed the experiments. Zhenxing Yang, Yuwen He, Teng He, Yongkang Li, Zhenxing Zhang, Susheng Li, Jinxin Meng, and Nan Li performed the experiments. Zhenxing Yang and Yuwen He conducted the data analysis. Zhenxing Yang and Haisheng Miao drafted and substantively revised the manuscript.

## Funding

This study was financially supported by the National Key Research and Development Program of China (Grant 2024YFD1800101), Guangxi Key Research and Development Project (Grant AB25069499), and Yunnan Fundamental Research Projects (Grant 202501AT070019).

## Disclosure

All authors contributed to the preparation of the article and approved the final submitted version.

## Ethics Statement

This article does not include studies involving human participants or animals conducted by the authors. The Institute for Yunnan Animal Science and Veterinary Institute, Kunming, China, authorized the collection of *Culicoides* and domestic animal serum samples.

## Conflicts of Interest

The authors declare no conflicts of interest.

## Supporting Information

Additional supporting information can be found online in the Supporting Information section.

## Supporting information


**Supporting Information 1** Table S1: The background information and GenBank accession numbers of all virus strains used in this study.

**Supporting Information 2** Table S2: Primers and probes for BTV serotype‐specific RT‐qPCR assays.

**Supporting Information 3** Table S3: Lengths of dsRNA segments 1–10, encoded putative proteins, 5′ and 3′ NCRs of the four BTV strains.

## Data Availability

The complete genome sequences of strains JH25C064, JH25C132, SZ25C057, and SJ25C081 from this study have been submitted to GenBank under Accession Numbers PZ244762–PZ244771, PZ244772– PZ244781, PZ244792–PZ244801, and PZ244782–PZ244791, respectively.
