# Supplementary material for: Bluetongue Virus Surveillance in Yunnan, China, 2025: Isolation of Multiple Serotypes From Culicoides and Seasonal Seroepidemiology in Cattle
Source: Transbound Emerg Dis. 2026 Jul 15;2026:8145184. doi: 10.1155/tbed/8145184 (PMC13370200; doi:10.1155/tbed/8145184)
Supplement: Supplementary file 2 — Supporting Information 2 Table S2: Primers and probes for BTV serotype‐specific RT‐qPCR assays. [file TBED-2026-8145184-s003.docx]

**Table S2: Primers and probes used in 12 serotype-specific RT-qPCR assays for BTV.**

| Serotypes | Names of primers and probes | Sequence (5'→3') | Location | Amplicon length (bp) |
| --- | --- | --- | --- | --- |
| BTV-1 | BTV-1_F | GGTTATGATGGGAARCGAGC | 261-281 | 197 |
|  | BTV-1_R | GTATCCGCTTTCTTTGARTCAATT | 415-435 |  |
|  | BTV-1_Probe | FAM-TCTTTCATCATCCAYTGTACCCATCGCTC-BHQ1 | 320-349 |  |
| BTV-2 | BTV-2_F | TAATACATTGGGAGTACCAGTTG | 1288-1311 | 156 |
|  | BTV-2_R | CATYTATTATCTCCCCRACCAT | 1400-1422 |  |
|  | BTV-2_Probe | FAM-CTATCACGTCCTCATCGCTRTCTG-BHQ1 | 1364-1389 |  |
| BTV-3 | BTV-3_F | ATTTACCGATTACRCACCCCGT | 2597-2619 | 97 |
|  | BTV-3_R | CTYCGTTTAAGCCTATCTATGCG | 2648-2671 |  |
|  | BTV-3_Probe | FAM-CGCGTCTGAAACCTCRAAAGCAACCAAAC-BHQ1 | 2623-2651 |  |
| BTV-4 | BTV-4_F | GTGCCGAGATTATTGGATATAAC | 236-259 | 134 |
|  | BTV-4_R | GGTTGTATATCCATYCGATCGTC | 324-347 |  |
|  | BTV-4_Probe | FAM-TCGCCCACTTCATCCACTTCGCATTA-BHQ1 | 299-325 |  |
| BTV-5 | BTV-5_F | ACTAGTGAGCCGTTCCAGCC | 626-646 | 94 |
|  | BTV-5_R | TTGAAGTAAYCCTGCGCGGAA | 716-737 |  |
|  | BTV-5_Probe | FAM-ATACCCAGGATCAGCCATGTCAACCCT-BHQ1 | 683-710 |  |
| BTV-7 | BTV-7_F | AATATGTTTCCGTGCTTACGAG | 1093-1115 | 157 |
|  | BTV-7_R | GTGCGTACACGTARTTCTCATT | 1206-1238 |  |
|  | BTV-7_Probe | FAM-TGCGAGCGAAACTCAATTCGGCGAT-BHQ1 | 1124-1149 |  |
| BTV-9 | BTV-9_F | GTACTCACGTCGYATATGGAG | 1064-1084 | 130 |
|  | BTV-9_R | CGMATGGCGTACACTCCAATTA | 1172-1193 |  |
|  | BTV-9_Probe | FAM-TCCGCYGCAATCAACGTGCCCCG-BHQ1 | 1107-1129 |  |
| BTV-12 | BTV-12_F | GTTGGCGYATATGGTGGAGTAA | 1067-1088 | 75 |
|  | BTV-12_R | CRTCCCCTAGYTCCATCTCA | 1123-1144 |  |
|  | BTV-12_Probe | FAM-CGTAYCCCTGCTTACGAGGAACGAT-BHQ1 | 1091-1115 |  |
| BTV-15 | BTV-15_F | TTTAGTTCCRACAGAGAGAGG | 2272-2293 | 81 |
|  | BTV-15_R | CAATCTCCCARACTCCCATCA | 2332-2332 |  |
|  | BTV-15_Probe | FAM-AGCTCCCTCCCAACCATCGCTGG-BHQ1 | 2306-2328 |  |
| BTV-16 | BTV-16_F | GAGGGAACTTCGAGCGAACA | 821-840 | 259 |
|  | BTV-16_R | CCGTTATTTGTGTCCGACGC | 1060-1079 |  |
|  | BTV-16_Probe | FAM-CAACAACCGATCCGGAAAGAAACG-BHQ1 | 970-994 |  |
| BTV-21 | BTV-21_F | ATATTCACRTCRAAAGGCCAAAG | 981-1003 | 169 |
|  | BTV-21_R | ATGTCCARTACACRTCTCCAAG | 1128-1149 |  |
|  | BTV-21_Probe | FAM-TTGGTGGACGAATCCGTACCCATGT-BHQ1 | 1070-1094 |  |
| BTV-24 | BTV-24_F | TACCGCTTCTTTTYCTTATCCA | 2228-2249 | 120 |
|  | BTV-24_R | TCGCATAAGCTCCAACTTCAAC | 2326-2347 |  |
|  | BTV-24_Probe | FAM-TGGTCTGTCCCAGTGATACTWTATGGC-BHQ1 | 2278-2304 |  |
