# Supplementary material for: Bluetongue Virus Surveillance in Yunnan, China, 2025: Isolation of Multiple Serotypes From Culicoides and Seasonal Seroepidemiology in Cattle
Source: Transbound Emerg Dis. 2026 Jul 15;2026:8145184. doi: 10.1155/tbed/8145184 (PMC13370200; doi:10.1155/tbed/8145184)
Supplement: Supplementary file 3 — Supporting Information 3 Table S3: Lengths of dsRNA segments 1–10, encoded putative proteins, 5′ and 3′ NCRs of the four BTV strains. [file TBED-2026-8145184-s001.docx]

**Table S3: Lengths of dsRNA segments 1–10, encoded putative proteins, 5' and 3′ NCRs of the four BTV strains.**

| Strains | Segment | Protein encoded | Segment length (bp) | Size of protein (aa) | Protein molecular mass (kDa) | G+C content (%) | 5' NCR (bp) | Terminal sequence (5'–3') ^a^ | 3' NCR (bp) | Stop-codon | GenBank Accession no. |
| --- | --- | --- | --- | --- | --- | --- | --- | --- | --- | --- | --- |
| JH25C064 | Seg-1 | VP1 | 3944 | 1302 | 149.66 | 41.81 | 11 | **GUUAAA**AT---AC**ACUUAC** | 24 | UGA | PZ244762 |
|  | Seg-2 | VP2 | 2940 | 961 | 112.13 | 41.26 | 17 | **GUUAAA**AT---CC**ACUUAC** | 37 | UGA | PZ244763 |
|  | Seg-3 | VP3 | 2772 | 901 | 103.30 | 44.26 | 17 | **GUUAAA**TT---AC**ACUUAC** | 49 | UAG | PZ244764 |
|  | Seg-4 | VP4 | 1981 | 644 | 75.22 | 43.21 | 8 | **GUUAAA**AC---AA**ACUUAC** | 38 | UGA | PZ244765 |
|  | Seg-5 | NS1 | 1763 | 552 | 64.73 | 42.6 | 34 | **GUUAAA**AA---CA**ACUUAC** | 70 | UAG | PZ244766 |
|  | Seg-6 | VP5 | 1635 | 526 | 59.03 | 43.55 | 25 | **GUUAAA**AA---AC**ACUUAC** | 29 | UGA | PZ244767 |
|  | Seg-7 | VP7 | 1154 | 349 | 38.54 | 47.4 | 17 | **GUUAAA**AA---AC**ACUUAC** | 87 | UAG | PZ244768 |
|  | Seg-8 | NS2 | 1125 | 354 | 40.46 | 44.09 | 19 | **GUUAAA**AA---AC**ACUUAC** | 41 | UAG | PZ244769 |
|  | Seg-9 | VP6 | 1052 | 330 | 35.58 | 47.53 | 15 | **GUUAAA**AA---AC**ACUUAC** | 44 | UAA | PZ244770 |
|  | Seg-10 | NS3 | 822 | 229 | 25.50 | 45.01 | 19 | **GUUAAA**AA---AC**ACUUAC** | 113 | UAA | PZ244771 |
|  | Total | | 19188 | 6148 | 704.15 | 43.37 | 182 | **GUUAAA**------------**ACUUAC** | 532 |  |  |
| JH25C132 | Seg-1 | VP1 | 3944 | 1302 | 149.61 | 41.58 | 11 | **GUUAAA**AT---AC**ACUUAC** | 24 | UGA | PZ244772 |
|  | Seg-2 | VP2 | 2921 | 955 | 109.96 | 41.73 | 17 | **GUUAAA**AG---AC**ACUUAC** | 36 | UAG | PZ244773 |
|  | Seg-3 | VP3 | 2772 | 901 | 103.30 | 44.08 | 17 | **GUUAAA**TT---AC**ACUUAC** | 49 | UAG | PZ244774 |
|  | Seg-4 | VP4 | 1981 | 644 | 75.27 | 42.96 | 8 | **GUUAAA**AC---AA**ACUUAC** | 38 | UGA | PZ244775 |
|  | Seg-5 | NS1 | 1763 | 552 | 64.76 | 42.71 | 34 | **GUUAAA**AA---CA**ACUUAC** | 70 | UAG | PZ244776 |
|  | Seg-6 | VP5 | 1637 | 526 | 58.99 | 46.67 | 28 | **GUUAAA**AA---CC**ACUUAC** | 28 | UGA | PZ244777 |
|  | Seg-7 | VP7 | 1156 | 349 | 38.63 | 47.84 | 17 | **GUUAAA**AA---AC**ACUUAC** | 89 | UAG | PZ244778 |
|  | Seg-8 | NS2 | 1125 | 354 | 40.41 | 44.8 | 19 | **GUUAAA**AA---AC**ACUUAC** | 41 | UAG | PZ244779 |
|  | Seg-9 | VP6 | 1052 | 330 | 35.64 | 47.81 | 15 | **GUUAAA**AA---AC**ACUUAC** | 44 | UAA | PZ244780 |
|  | Seg-10 | NS3 | 822 | 229 | 25.51 | 45.99 | 19 | **GUUAAA**AA---AC**ACUUAC** | 113 | UAA | PZ244781 |
|  | Total | | 19173 | 6142 | 702.08 | 43.74 | 185 | **GUUAAA**------------**ACUUAC** | 532 |  |  |
| Strains | Segment | Protein encoded | Segment length (bp) | Size of protein (aa) | Protein molecular mass (kDa) | G+C content (%) | 5' NCR (bp) | Terminal sequence (5'–3') ^a^ | 3' NCR (bp) | Stop-codon | GenBank Accession no. |
| SZ25C057 | Seg-1 | VP1 | 3944 | 1302 | 149.79 | 41.89 | 11 | **GUUAAA**AT---AC**ACUUAC** | 24 | UGA | PZ244792 |
|  | Seg-2 | VP2 | 2926 | 956 | 110.16 | 41.73 | 19 | **GUUAAA**AG---AC**ACUUAC** | 36 | UAG | PZ244793 |
|  | Seg-3 | VP3 | 2772 | 901 | 103.30 | 43.9 | 17 | **GUUAAA**TT---AC**ACUUAC** | 49 | UAG | PZ244794 |
|  | Seg-4 | VP4 | 1981 | 644 | 75.27 | 43.56 | 8 | **GUUAAA**AC---AA**ACUUAC** | 38 | UGA | PZ244795 |
|  | Seg-5 | NS1 | 1763 | 552 | 64.75 | 42.6 | 34 | **GUUAAA**AA---CA**ACUUAC** | 70 | UAG | PZ244796 |
|  | Seg-6 | VP5 | 1638 | 526 | 59.25 | 45.67 | 29 | **GUUAAA**AA---AC**ACUUAC** | 28 | UGA | PZ244797 |
|  | Seg-7 | VP7 | 1154 | 349 | 38.55 | 47.49 | 17 | **GUUAAA**AA---AC**ACUUAC** | 87 | UAG | PZ244798 |
|  | Seg-8 | NS2 | 1125 | 354 | 40.45 | 43.82 | 19 | **GUUAAA**AA---AC**ACUUAC** | 41 | UAG | PZ244799 |
|  | Seg-9 | VP6 | 1052 | 330 | 35.70 | 47.72 | 15 | **GUUAAA**AA---AC**ACUUAC** | 44 | UAA | PZ244800 |
|  | Seg-10 | NS3 | 822 | 229 | 25.46 | 46.59 | 19 | **GUUAAA**AA---AC**ACUUAC** | 113 | UGA | PZ244801 |
|  | Total | | 19177 | 6143 | 702.67 | 43.69 | 188 | **GUUAAA**------------**ACUUAC** | 530 |  |  |
| SJ25C081 | Seg-1 | VP1 | 3944 | 1302 | 149.79 | 41.68 | 11 | **GUUAAA**AT---AC**ACUUAC** | 24 | UGA | PZ244782 |
|  | Seg-2 | VP2 | 2935 | 959 | 112.38 | 41.4 | 21 | **GUUAAA**AA---AC**ACUUAC** | 34 | UAA | PZ244783 |
|  | Seg-3 | VP3 | 2772 | 901 | 103.33 | 43.72 | 17 | **GUUAAA**TT---AC**ACUUAC** | 49 | UAG | PZ244784 |
|  | Seg-4 | VP4 | 1981 | 644 | 75.22 | 43.11 | 8 | **GUUAAA**AC---AA**ACUUAC** | 38 | UGA | PZ244785 |
|  | Seg-5 | NS1 | 1763 | 552 | 64.74 | 42.37 | 34 | **GUUAAA**AA---CA**ACUUAC** | 70 | UAG | PZ244786 |
|  | Seg-6 | VP5 | 1637 | 526 | 59.14 | 44.78 | 28 | **GUUAAA**AA---AC**ACUUAC** | 28 | UGA | PZ244787 |
|  | Seg-7 | VP7 | 1156 | 349 | 38.66 | 45.85 | 17 | **GUUAAA**AA---AC**ACUUAC** | 89 | UAG | PZ244788 |
|  | Seg-8 | NS2 | 1125 | 354 | 40.55 | 44.8 | 19 | **GUUAAA**AA---AC**ACUUAC** | 41 | UAG | PZ244789 |
|  | Seg-9 | VP6 | 1052 | 330 | 35.81 | 47.53 | 15 | **GUUAAA**AA---AC**ACUUAC** | 44 | UAA | PZ244790 |
|  | Seg-10 | NS3 | 822 | 229 | 25.46 | 46.59 | 19 | **GUUAAA**AA---AC**ACUUAC** | 113 | UAA | PZ244791 |
|  | Total | | 19187 | 6146 | 705.08 | 43.37 | 189 | **GUUAAA**------------**ACUUAC** | 530 |  |  |

^a^ Conserved nucleotide sequences in 5′- and 3′-terminals are shown in bold.
